# Recent Advances in Mass Spectrometry-Based Studies of Post-Translational Modifications in Alzheimer’s Disease

**DOI:** 10.1016/j.mcpro.2025.101003

**Published:** 2025-05-29

**Authors:** Feixuan Wu, Wei Li, Haiyan Lu, Lingjun Li

**Affiliations:** 1 School of Pharmacy, University of Wisconsin-Madison, Madison, Wisconsin, USA; 2 Department of Chemistry, University of Wisconsin-Madison, Madison, Wisconsin, USA; 3 Biophysics Graduate Program, University of Wisconsin-Madison, Wisconsin, USA; 4 Lachman Institute for Pharmaceutical Development, School of Pharmacy, University of Wisconsin-Madison, Madison, Wisconsin, USA; 5 Wisconsin Center for NanoBioSystems, School of Pharmacy, University of Wisconsin-Madison, Madison, Wisconsin, USA

**Keywords:** Alzheimer's disease, citrullination, glycosylation, Post-translational modifications, phosphorylation

## Abstract

Alzheimer’s disease (AD) is a progressive neurodegenerative disorder characterized by cognitive decline. There are more than 10 million new cases of AD each year worldwide, implying one new case every 3.2 s. Post-translational modifications (PTMs) such as phosphorylation, glycosylation, and citrullination have emerged as key modulators of protein function in AD, influencing protein aggregation, clearance, and toxicity. Mass spectrometry (MS) has become an indispensable tool for detecting and quantifying these PTMs, offering valuable insights into their role in AD pathogenesis. This review explores recent advancements in MS-based studies of PTMs in AD, with emphasis on MS techniques, such as data-dependent acquisition (DDA) and data-independent acquisition (DIA), as well as enrichment methods used to characterize PTMs. The applications of these MS-based approaches to the study of various PTMs are highlighted, which have significantly accelerated the biomarker discovery process, providing new avenues for early diagnosis and therapeutic targeting. Advances in biological understanding and analytical techniques, while addressing the challenges and future directions, will be discussed.

## Highlights

• Comprehensive review of recent mass spectrometry advancements for studying post-translational modifications in AD.
• Key focus on phosphorylation, glycosylation, and citrullination and their roles in AD pathology and biomarker discovery.
• Novel mass spectrometry techniques enable site-specific PTM analysis, offering insights into therapeutic targets for AD.

## Graphical Abstract

**Figure:**
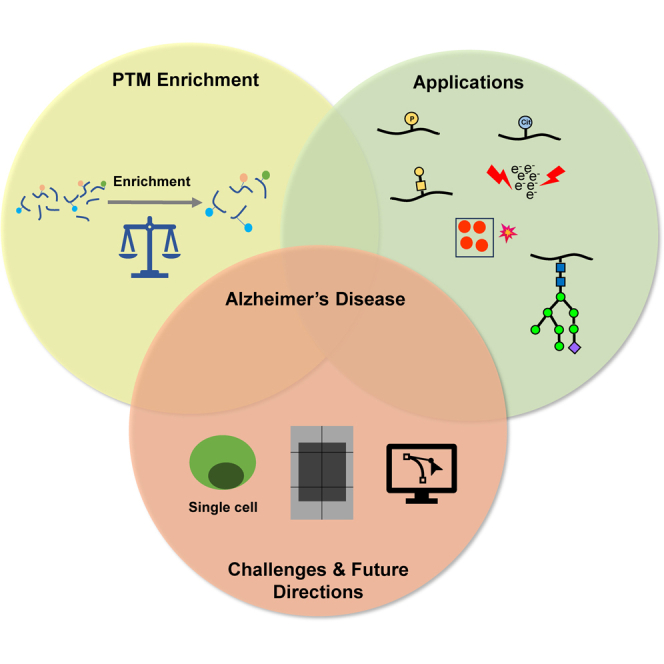

## In Brief

This review provides a comprehensive overview of recent advancements in mass spectrometry (MS)-based techniques for investigating post-translational modifications (PTMs) in Alzheimer’s disease (AD). With a focus on phosphorylation, glycosylation, citrullination, and other critical PTMs, we highlight how these modifications influence the aggregation, clearance, and toxicity of key proteins like tau and amyloid precursor protein (APP) involved in AD pathogenesis. By leveraging novel MS techniques, this review highlights the potential of PTM-targeted biomarkers and therapeutic strategies in AD, offering new insights into the molecular mechanisms driving the disease and opening new avenues for early diagnosis and treatment.

Alzheimer's disease (AD) is a progressive neurodegenerative disorder characterized by cognitive decline, memory loss, and behavioral changes, ultimately leading to severe dementia (1). In most patients suffering from AD, symptoms first appear later in life (1, 2). As of 2023, it is estimated that over 55 million people worldwide are living with AD or other forms of dementia (2). This number is expected to rise significantly due to an increase in aging populations, with projections suggesting that it could reach 139 million by 2050 (2). Pathologically, AD is marked by the accumulation of amyloid-beta (Aβ) plaques and neurofibrillary tangles composed of hyperphosphorylated tau proteins in the brain (3). These hallmark features, along with neuroinflammation and synaptic dysfunction, contribute to the gradual loss of neurons and synapses, which underlie the clinical symptoms of the disease (2). Despite extensive research, the exact etiology of AD remains elusive, and current treatments provide only symptomatic relief without halting disease progression. This underscores the urgent need for a deeper understanding of the molecular mechanisms driving AD pathogenesis to develop more effective therapeutic strategies.

Protein PTMs are increasingly recognized as key contributors to pathology of AD. For instance, the hyperphosphorylation of tau protein leads to the formation of neurofibrillary tangles (4). Other PTMs, such as glycosylation, citrullination, and acetylation, also modulate the aggregation, clearance, and toxicity of proteins involved in AD, including amyloid precursor protein (APP) and Aβ (5, 6). These modifications can influence the progression of AD by altering protein conformations and interactions, thereby impacting cellular signaling pathways and neuronal health (7, 8). Understanding the role of PTMs in AD could provide valuable insights into the investigation of biochemical signaling pathways during AD pathogenesis. Traditional methods for detecting PTMs include techniques such as western blotting (9), immunoprecipitation (10), and radiolabeling (11). These approaches have been widely used due to their relative simplicity and the availability of modification-specific antibodies. Western blotting, for instance, offers the advantage of being straightforward and cost-effective, allowing for the detection of specific PTMs in a semi-quantitative manner. Immunoprecipitation facilitates the enrichment of modified proteins using modification-specific antibodies, while radiolabeling techniques have historically provided a sensitive method for detecting PTMs like phosphorylation (11). However, these traditional methods come with significant limitations. One major drawback is their dependency on high-quality, modification-specific antibodies, which are not always available for less studied or novel PTMs. Moreover, these techniques generally provide limited information on the exact sites of modification and often lack the sensitivity required to detect low-abundance modifications. Their capacity for large-scale or high-throughput analysis is also restricted, making them less suitable for comprehensive proteomic studies.

In contrast, MS offers several advantages that overcome these limitations. MS is a powerful analytical technique used to identify and quantify molecules based on their *m/z* ratio (12). It has emerged as an indispensable tool in PTM studies due to its high sensitivity, accuracy, and ability to analyze complex protein modifications (13). MS-based approaches enable site-specific identification of a wide range of PTMs in a single experiment without the need for modification-specific reagents. These techniques facilitate the identification of PTM sites, the stoichiometry of modifications, and the dynamics of these modifications under various conditions, including disease states such as AD (14, 15, 16, 17). MS has been instrumental in uncovering the PTMs associated with key proteins involved in AD, providing insights into their functional consequences and contributions to neurodegeneration. Here we discuss the recent advancements of MS applications in PTM in AD, including recent advances in MS-based methods for identifying PTMs, the specific PTMs associated with AD, and how these modifications impact AD pathology, and offer potential avenues for the development of novel biomarkers and therapeutic targets.

## MS Techniques for PTM Detection

**Fig. 1:**
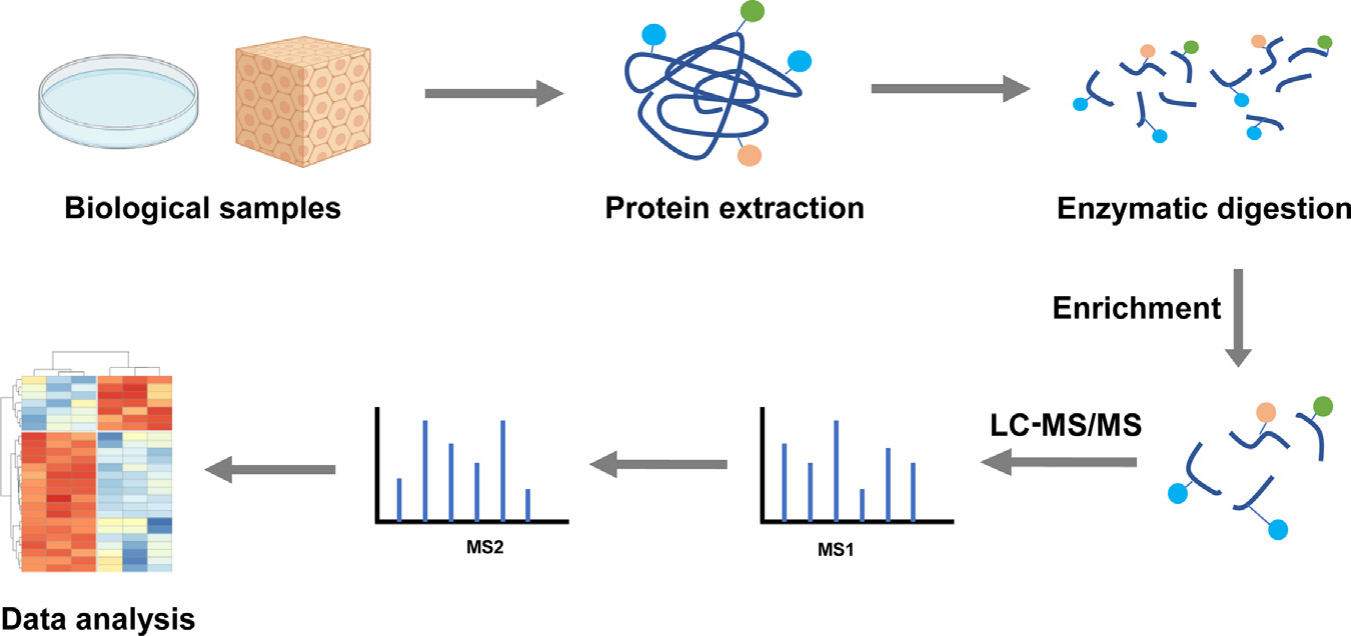
**The workflow of bottom-up PTM analysis.** Proteins are extracted from various biological samples followed by enzymatic digestion. The peptides are enriched prior to LC-MS/MS and the data are then processed using appropriate tools and analyzed for visualization.

MS has emerged as a central technology for identifying and characterizing PTMs owing to its exceptional sensitivity, precision, and capability to analyze complex biological samples. MS offers two primary approaches for protein analysis: the top-down method, which examines intact proteins (18, 19), and the more widely used bottom-up approach, which analyzes peptides generated through proteolysis (20, 21). The bottom-up approach is integral to proteomics, enabling protein identification, PTM characterization, and quantification. The main steps of MS experiments are shown in Figure 1. The analytes of interest are initially extracted from biological samples, including cells, tissues, and biofluids. Due to the low abundance of PTMs and inefficient ionization, enrichment is frequently required. The choice of enrichment strategies depends on specific PTM, as discussed in the following section. Following enrichment, peptides are analyzed using liquid chromatography-tandem mass spectrometry (LC-MS/MS). This approach can be performed using one of two acquisition methods: DDA (22, 23), where precursor ions are selectively isolated based on their relative abundances, and DIA, where all ions within specified *m/z* windows are fragmented without prior selection (24).

MS enables the determination of over 400 different types of PTMs to date (25), many of which play crucial roles in AD pathology. This review examines the key PTMs relevant to AD, including phosphorylation (+79.966 Da), glycosylation (≥203 Da), acetylation (+42.010 Da), citrullination (+0.985 Da), S-nitrosylation (+29.998 Da), methylation (+14.016 Da), SUMOylation (+600.250 Da and +599.266 Da), and ubiquitination (Gly-Gly remnant left after tryptic digestion of ubiquitinated lysine residues, +114.0429 Da) (25, 26, 27, 28), highlighting their target amino acid residues, biological functions, and their contributions to AD pathology. These modifications play crucial roles in regulating protein functionality and related signaling pathways, and their dysregulation is often implicated in AD progression. However, due to the low abundance of PTMs, enrichment is required to enable more sensitive detection (29). Table 1 outlines various enrichment methods for most AD-related PTMs.

**Table 1:** Overview of the enrichment methods for AD related PTMs

PTMs	Targeted residues	Main biological functions	Enrichment methods
Phosphorylation	Ser, Thr, Tyr	signal transduction, enzyme activation/inhibition, cell cycle regulation, protein-protein interactions	IMAC, MOAC, HILIC, ERLIC, aminoxy, boronic acid, bioorthogonal chemical labeling, bioorthogonal metabolic labeling
Glycosylation	Asn (N-linked); Ser, Thr, Tyr (O-linked)	protein stability, cell-cell recognition, immune response, protein trafficking	lectins, modified glycosidases, immunoaffinity, IMAC, HILIC
Acetylation	Lys, Ser, Thr, N terminus	gene expression regulation, protein stability, cellular stress response, chromatin remodeling	immunoaffinity, SCX, ZIC-HILIC, COFRADIC, antibody-IEF, antibody-SCX
Citrullination	Arg	gene expression modulation, immune response, protein-protein interactions, chromatin remodeling	immunoaffinity, phenylglyoxal labeling, biotin thiol labeling
S-Nitrosylation	Cys, Tyr, Trp, Met, Lys	regulation of protein function, signal transduction, oxidative stress response, modulation of enzyme activity	immunoaffinity, bioorthogonal chemical labeling, biotin-switch
Methylation	Lys, Arg, His, Ala, Asn, Glu, Asp,	gene expression regulation, chromatin structure modulation, protein interactions, gene silencing	immunoaffinity, IEF, SCX, HILIC, antibody-SCX, antibody-HpH RP, 3xMBT methyl-binding domains, antibody-propionylation
Sumoylation	Lys	protein stability, nuclear localization, transcriptional regulation, protein-protein interactions	tagged-SUMO, immunoaffinity, SUMO-interacting motifs
Ubiquitylation	Met, Lys, N terminus, Cys, Ser, Thr	protein degradation, cell cycle regulation, DNA repair, signal transduction	tagged-Ub, immunoaffinity, COFRADIC

Phosphorylation is the addition of a phosphate group, typically to serine, threonine, or tyrosine residues, catalyzed by kinases (30). It plays a key role in regulating signal transduction pathways and cellular processes such as cell division and metabolism. Enrichment strategies for phosphopeptides commonly include metal oxide affinity chromatography (MOAC), such as titanium dioxide (TiO_2_) and immobilized metal affinity chromatography (IMAC), which selectively binds to phosphorylated residues (31, 32, 33, 34, 35, 36, 37). These methods are effective for enriching phosphorylated peptides. IMAC uses immobilized metal ions (e.g., Fe^3+^ and Ti^4+^) to bind phosphorylated residues, while MOAC uses metal oxides like TiO_2_. Advantages include high specificity for phosphorylated peptides, but the limitation is potentially binding to non-phosphorylated peptides with acidic residues. IMAC and MOAC provide strong enrichment for phosphopeptides but may suffer from non-specific binding (31). Combining it with other strategies (e.g., high-pH fractionation) increases peptide identification and coverage.

**Fig. 2:**
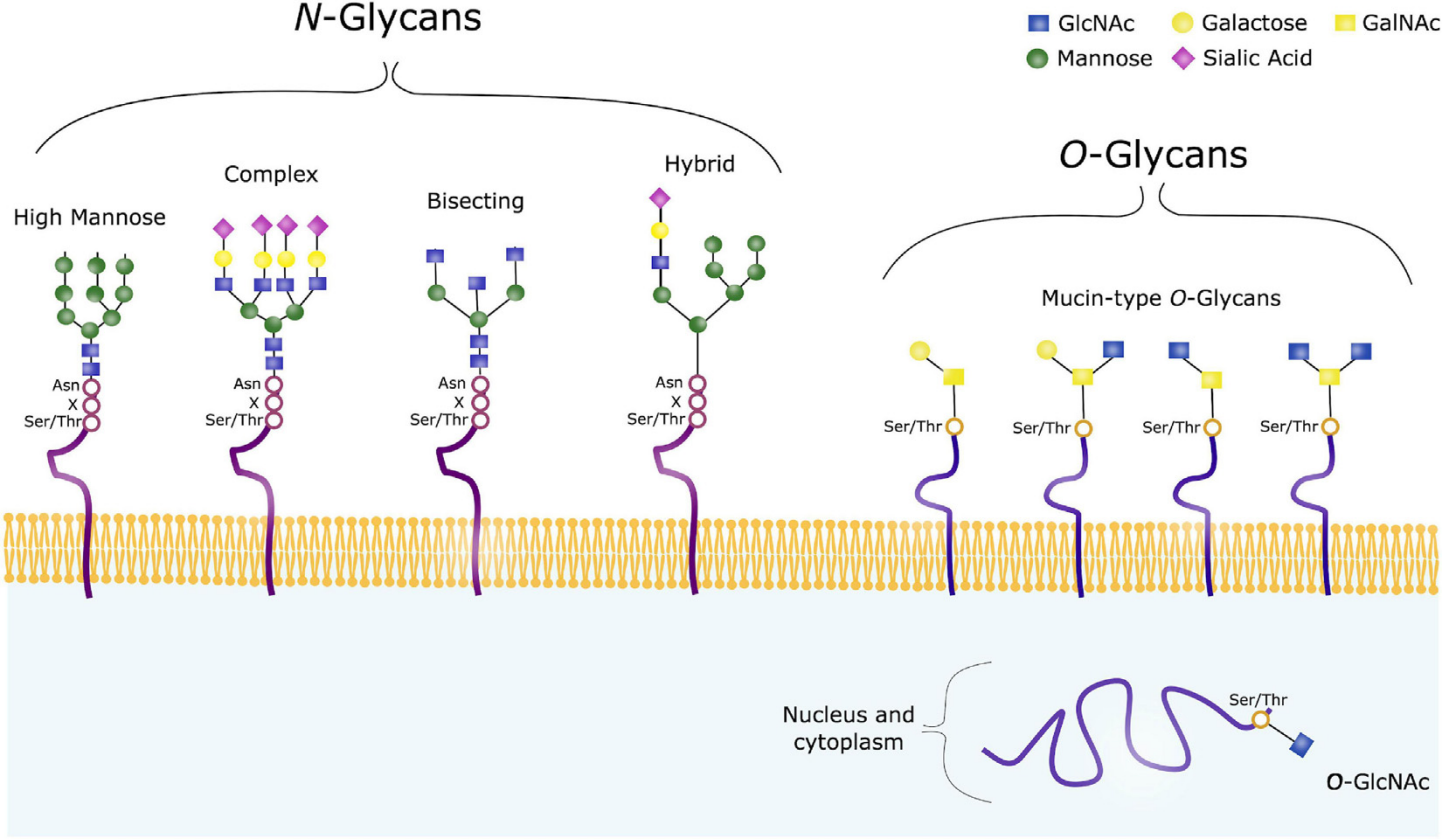
**Common Glycan Structures.** N-glycans share a common core structure composed of GlcNAc and mannose, which can be further trimmed and modified with sugars such as galactose, fucose, or sialic acids. The major types of N-glycans include high-mannose, complex, hybrid, and bisected structures. In contrast, O-glycans have diverse core structures, primarily based on either GalNAc or GlcNAc. O-GalNAc glycans, also known as mucin-type O-glycans, are commonly found on membrane-bound, extracellular, and secreted proteins. O-GalNAcylation is a complex form of O-glycosylation, with four major core structures illustrated. Meanwhile, O-GlcNAc modifications typically occur on intracellular proteins. Adapted from Haukedal *et al.* (40) with permission.

Glycosylation is a critical PTM where sugar molecules are covalently attached to proteins, which can occur as *N*-glycosylation (on Asn residues) or *O*-glycosylation (on Ser/Thr residues) (Fig. 2). *N*-glycosylation involves the attachment of a *N*-acetylglucosamine (GlcNAc) to the peptide backbone through a β-1N linkage. The process is initiated in the endoplasmic reticulum (ER), where the glycan precursor is transferred to proteins containing the consensus sequence Asn-X-Ser/Thr. Once attached, the glycan undergoes sequential trimming by mannosidases and glucosidases within the ER, followed by further modifications in the Golgi apparatus to ensure glycan maturity (38).

In contrast, *O*-glycosylation predominantly involves GlcNAc or *N*-acetylgalactosamine (GalNAc) modifications. GalNAc-linked *O*-glycans, often referred to as mucin-type *O*-glycans, are processed within the Golgi compartments. Unlike *N*-glycosylation, which involves distinct pre- and post-processing steps, *O*-glycan chain elongation occurs through the sequential action of specific glycosyltransferases, which add galactose, GlcNAc, sialic acid, or fucose residues. These *O*-glycans are predominantly found on extracellular and secreted glycoproteins. Meanwhile, intracellular glycoproteins primarily contain GlcNAc-linked *O*-glycans, where the biosynthesis occurs within the cytoplasm and is regulated by *O*-linked GlcNAc transferase (OGT) and *O*-GlcNAcase (OGA). Unlike mucin-type *O*-glycans, *O*-GlcNAcylation involves the addition of a single GlcNAc residue to Ser or Thr, without further sugar chain elongation. Given its dynamic and reversible nature, *O*-GlcNAcylation is closely linked to cellular signaling, stress response, and metabolic regulation (39, 40). Both *N*- and *O*-glycosylation significantly influence protein function, structure, stability, oligomerization, and aggregation, and it plays a crucial role in biological processes such as cell adhesion, communication, and host-pathogen interactions (41, 42, 43). To facilitate *O*-glycoproteomics analysis, endoglycosidases, such as peptide *N*-glycosidase (PNGase F), are often used to remove *N*-glycans (44, 45). This step helps reduce interference from *N*-glycans, allowing for more specific enrichment and detection of *O*-glycopeptides.

Common enrichment strategies for glycopeptides include lectin affinity chromatography, which uses lectins to bind specific carbohydrate structures (46, 47, 48), and hydrophilic interaction chromatography (HILIC), which exploits the hydrophilic properties of glycans (49, 50). Lectin-based enrichment relies on the recognition of specific monosaccharides or glycan motifs rather than entire glycan structures. While certain lectins, such as Concanavalin A (ConA) and *Phaseolus vulgaris* leucoagglutinin (PHA-L), preferentially bind to N-glycans (e.g., high-mannose and complex-type N-glycans) (51), others, like Jacalin and *Vicia villosa* agglutinin (VVA), are more selective for O-glycans (e.g., Galβ1-3GalNAc) (52). HILIC, on the other hand, enriches a broader range of glycosylated peptides. While lectins offer high specificity, they can miss certain glycoforms, and HILIC may co-enrich non-glycosylated hydrophilic peptides. More enrichment strategies and discussions can be found in Riley *et al.* (53).

Acetylation refers to the addition of an acetyl group, typically to lysine residues, and is a key regulator of gene expression, protein function, and chromatin structure (54). Acetylated peptides exhibit differences in hydrophobicity and charge compared to their non-acetylated counterparts, which can facilitate their separation using various chromatographic techniques. While reversed-phase high-performance liquid chromatography (RP-HPLC) can distinguish individual acetylated peptides from their non-acetylated forms, its effectiveness is limited in complex mixtures due to the relatively small retention time shifts caused by acetylation. Strong cation exchange chromatography (SCX) leverages differences in charge states, offering an alternative means of enrichment, while zwitterionic hydrophilic interaction liquid chromatography (ZIC-HILIC) can further enhance separation by combining both hydrophobic and electrostatic interactions (55, 56). Additionally, HILIC provides the advantage of being able to separate methylated and acetylated histones, thus reducing sample complexity (57). Moreover, combined fractional diagonal chromatography (COFRADIC) combines sequential chromatographic steps, typically involving charge-based and chemical modifications, to distinguish acetylated peptides from their non-acetylated counterparts (58). Immunoprecipitation with acetylation-specific antibodies ensures high specificity, but antibody availability and cost can limit its routine use (59).

Citrullination involves the conversion of arginine residues to citrulline by the enzyme peptidylarginine deiminase (PAD) (60). This modification alters the charge and structure of proteins, influencing their function and interactions (60). Citrullination plays a crucial role in various physiological processes, including gene expression regulation, immune response, protein-protein interactions, and chromatin remodeling. Enrichment of citrullinated peptides is challenging due to their low abundance and structural similarity to unmodified peptides. Affinity-based methods using citrulline-specific antibodies and chemical derivatization approaches have been developed to improve their detection (61, 62, 63, 64, 65, 66). The latter approach is particularly useful for citrullination, where direct enrichment is difficult. Chemical derivatization, such as biotin thiol labeling, improves the selectivity of MS detection but can introduce complexity and variability in the workflow.

## MS Application in PTM-Related Drug Development for AD

The complexity of AD pathology necessitates the use of high-resolution proteomics to comprehensively analyze global PTM changes associated with disease progression and therapeutic interventions. MS-based approaches, such as quantitative phosphoproteomics, glycoproteomics, and intact PTM profiling, have emerged as essential tools for elucidating drug-target interactions and the mechanistic effects of therapeutic agents (67, 68). One of the key applications of MS in AD drug development is target identification and validation, where it enables the systematic identification of disease-relevant PTMs, including tau phosphorylation, Aβ glycosylation, and citrullination-related immune dysregulation (69, 70). These modifications play critical roles in AD pathogenesis and serve as key therapeutic targets for drug discovery efforts.

Here are several examples of drugs targeting PTMs for AD treatment. Glycogen synthase kinase-3 (GSK-3) inhibitors (71), including Tideglusib from Zeltia, which completed Phase II trials (72); lithium, developed by multiple companies (73); and SAR502250 from Sanofi (74). GSK-3 is an enzyme implicated in tau protein hyperphosphorylation. Hyperphosphorylated tau aggregates into neurofibrillary tangles, disrupting neuronal function and contributing to neurodegeneration. Inhibiting GSK-3 aims to reduce this pathological phosphorylation, stabilizing tau and potentially slowing disease progression. Histone deacetylase (HDAC) inhibitors represent another promising class of therapeutics, including Entinostat in Phase II clinical trials from MedImmune (a subsidiary of AstraZeneca) (75), Trichostatin A (TSA) (76), and Vorinostat (SAHA) (77). These inhibitors increase tau acetylation, which may reduce tau aggregation and mitigate neurodegeneration. By blocking HDAC activity, they promote a more acetylated and less aggregation-prone form of tau. OGA inhibitors are also under investigation, such as Thiamet G in preclinical stages from Medifron DBT (78, 79), and Ceperognastat in Phase I clinical trials from Eli Lilly (80). These compounds aim to elevate tau O-GlcNAcylation levels, a modification that may protect tau from hyperphosphorylation and aggregation, thereby offering a potential therapeutic avenue in AD.

Additionally, mechanism-of-action studies benefit from advanced MS techniques such as targeted multiple reaction monitoring (MRM) and DIA-MS, which provide quantitative assessments of PTM alterations in response to drug treatments, thereby ensuring mechanistic validation of therapeutic compounds (1, 81). Beyond target identification and mechanistic studies, MS has also been integrated into clinical trials to facilitate biomarker discovery and validation. Biomarkers such as pTau181, pTau217, and the Aβ42/Aβ40 ratio, identified through MS-based proteomics, are being used to monitor drug efficacy and track disease progression in clinical settings (82, 83, 84). These biomarkers have proven valuable for patient stratification and treatment response assessment, reinforcing the role of MS as a powerful tool in precision medicine approaches for AD drug development.

## Applications

### Phosphorylation

Protein phosphorylation by kinases and dephosphorylation by phosphatases is a crucial PTM that regulates numerous cellular processes, including protein activity, localization, and interactions (85, 86). In AD, abnormal phosphorylation, particularly of tau protein, is a hallmark of the disease. Hyperphosphorylated tau undergoes conformational changes, promoting oligomerization and fibril formation, which are hallmarks of aggregates to form neurofibrillary tangles (87). Moreover, the phosphorylation of APP and other proteins associated with AD has been implicated in the disease’s progression (88). Nevertheless, the ever-changing nature of signaling networks, the intricate complexity of the phosphoproteome, and the low stoichiometry of protein phosphorylation present significant technical challenges (89). MS has emerged as the best analytical tool for phosphorylation identification and quantification, providing invaluable insights into the potential diagnostic or therapeutic relevance underlying AD.

**Fig. 3:**
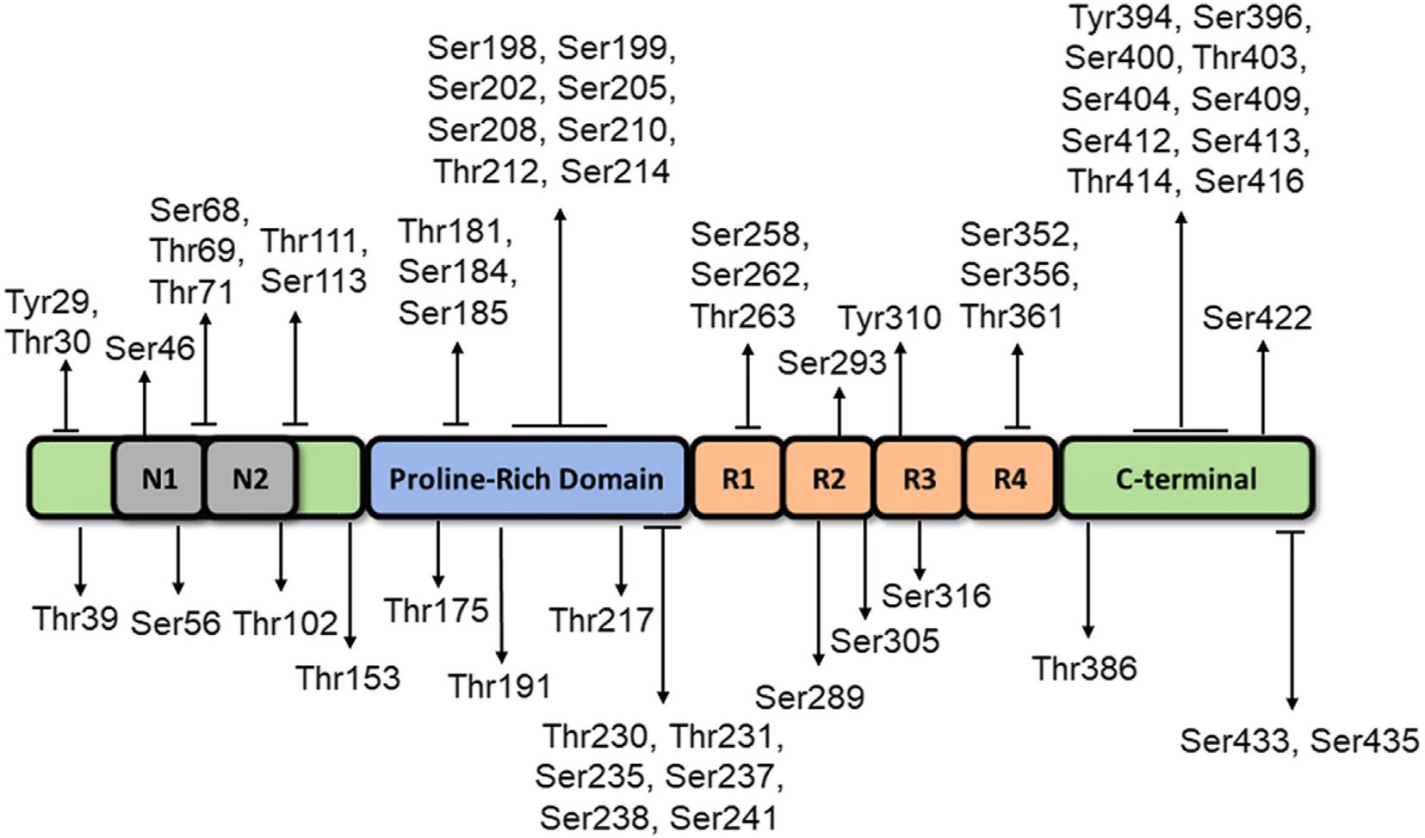
**Aschematicdiagramshowing 2N4R tau (441 amino acids), the longest isoform expressed in human brain.** Tau protein contains major structural domains including N-terminal domain with N1 and N2 inserts, proline rich region, four major microtubule-binding repeats (R1-R4), and C-terminal domain. The N1, N2 and R2 regions can be alternatively spliced in the human brain resulting in 6 isoforms: 0N3R, 1N3R, 2N3R, 0N4R, 1N4R, and 2N4R. The position of identified phosphorylation sites found in AD brains are shown. Adapted from Xia *et al.* (101) with permission.

Tau, a microtubule-associated protein present in neurons and glial cells, plays a crucial role in cytoskeletal stabilization (90). Its primary structure characterized by its interaction with microtubules, and its amino acid composition can be segmented into distinct regions: an N-terminal projection domain, a proline-rich region, a repeat region, and a C-terminal domain. In the human brain, tau exists in six splice variants, which differ in the number of N-terminal inserts and the presence or absence of one of the four repeats (R1-R4) within the microtubule-binding domain, specifically the second repeat (R2) (91). Structural alteration in tau, whether due to mutations or PTMs, particularly specific phosphorylation events, diminishes its affinity for microtubules. This reduction in binding promotes the accumulation and aggregation of tau into toxic species, such as neurofibrillary tangles and paired helical filaments (92). The phosphorylation stoichiometry of Tau in pathological aggregates is approximately three times higher than that of the physiological, soluble form of Tau (93), and this hyperphosphorylation is believed to be a critical step in the pathogenesis of AD (94). Recent advancements in MS, particularly in MS/MS, have enabled the detailed characterization of tau phosphorylation sites (95, 96, 97). Studies have identified more than 80 phosphorylation sites in the longest isoform of tau, with many located within the proline-rich region and the C-terminal microtubule-binding domain (7, 98, 99, 100) (Fig. 3). For instance, phosphorylation at Ser202, Thr205, and Ser396/404 has been consistently associated with tau pathology in AD (7, 101). Notably, hyperphosphorylation of tau is closely associated with tau aggregation and pathology in AD (93). Wisniewski and colleagues identified 23 distinct phosphorylation sites on the tau protein using LC-MS analysis of brain tissues from seven patients with advanced sporadic AD (102), with Ser369 being the most abundantly phosphorylated. Later, Bateman *et al.* expanded the identification of phosphorylated tau sites in the human brain to 29 distinct sites (103). Of these, 12 sites were also detected in CSF samples from AD patients. Moreover, a recent study from the Dominantly Inherited Alzheimer Network (DIAN) cohort quantified site-specific phosphorylation of tau in CSF samples from AD patients across 4 decades of disease progression (104). They proposed a tau staging model, where phosphorylation changes in tau occur at different stages of AD, following distinct temporal trajectories. Notably, these two isoforms, pTau217 and pTau181, can be detected in CSF of patients with AD up to 2 decades prior to the onset of pathological tau aggregation (104). Similarly, Jacquemin *et al* employed the single-pot, solid phase-enhanced sample-preparation (SP3) protocol to achieve antibody-free quantification of pathological tau from AD brain sample. This approach enabled the identification and quantification of tau peptides, including peptides representative of two isoforms and two phosphopeptides, pTau217 and pTau181 (105). These findings suggest that the identification of early-stage phosphorylation events may serve as potential biomarkers for early diagnosis. Additionally, Blennow and colleagues developed a MS method to simultaneously quantify tau peptides in plasma, indicating that pTau217, pTau231 and pTau205 are the plasma tau forms that best reflect AD-related brain pathological changes and further supporting its potential as a diagnostic tool (96, 106, 107). Notably, pTau181 and pTau217 are FDA-approved CSF and plasma biomarkers for AD diagnosis, with pTau217 demonstrating superior diagnostic performance in correlating with amyloid PET imaging and predicting preclinical AD (82, 83).

APP is another critical protein in AD, and its phosphorylation plays a significant role in the generation of Aβ peptides, the primary components of amyloid plaques (88). Among Aβ fragments, Aβ1-40 and Aβ1-42 are the most extensively studied due to their distinct biophysical properties and pathological implications (108). Aβ1-40 constitutes the majority of Aβ peptides produced in the brain under physiological conditions (109). While it is less prone to aggregation than Aβ1-42, it is a key component in cerebral amyloid angiopathy, a condition characterized by the deposition of Aβ in the walls of cerebral blood vessels (110). In contrast, Aβ1-42, though less abundant, is highly hydrophobic and aggregation-prone, forming oligomers, protofibrils, and amyloid plaques in brain parenchyma (111). Studies suggest that soluble Aβ1-42 oligomers, rather than mature fibrils, are the most neurotoxic species, as they disrupt synaptic function, impair long-term potentiation, and induce neuroinflammation (112). Aβ42/Aβ40 ratio in CSF has also been FDA-approved as an early AD biomarker. MS techniques have enabled the detection of phosphorylated Aβ peptides in both *in vitro* and *in vivo* settings. Notably, phosphorylation at Ser8 has been identified in human CSF, with studies indicating that this modification enhances Aβ aggregation and may reflect the severity of amyloidosis (113). Furthermore, the development of specialized matrices, such as TOPAC, has improved the sensitivity of MS in detecting intact phosphorylated Aβ species, addressing challenges related to sample preparation and analysis (114).

Beyond Aβ phosphorylation, APP itself undergoes phosphorylation, which influences its processing and Aβ production. The cytoplasmic domain of APP contains eight potential phosphorylation sites, specifically Thr654, 668, and 686; Ser655 and 675; and Tyr653, 682, and 687, according to the APP695 isoform numbering (115). Seven of the eight phosphorylation sites, except for Thr654, have been detected in the brains of AD patients (116), indicating that APP phosphorylation may play a crucial role in the physiological function and processing of APP within the central nervous system. High-resolution MS, combined with phosphopeptide enrichment techniques, has allowed the identification of phosphorylation sites on APP and its fragments. The phosphorylation of APP at Thr668 has garnered significant attention due to its widespread occurrence in AD and its substantial impact on APP cleavage (117). For example, phosphorylation at Thr668 has been shown to promote the cleavage of APP by beta-secretase (BACE1), resulting in the production of Aβ (116). This site-specific phosphorylation can be quantitatively analyzed using MS, providing insights into the molecular mechanisms that contribute to Aβ accumulation in AD (118). Lannuzzi *et al* also found the possibility that overactivation of Fyn enhances phosphorylation of APP at Tyr682, initiating amyloidogenic processing of APP in neurons from patients with AD (119). Recently, Drummond *et al* showed that phosphorylated Aβ is significantly higher in Down Syndrome with early onset AD through label-free quantification and fluorescent immunohistochemistry (120). These studies have revealed potential therapeutic targets that could be modulated to reduce Aβ production and accumulation in AD.

Given its central role in tau and APP pathology, modulating phosphorylation has emerged as a promising therapeutic approach. Targeting kinases responsible for tau hyperphosphorylation has been explored in preclinical and clinical trials. Several kinase inhibitors have been developed to reduce pathological tau phosphorylation. Among the most investigated kinases, GSK3β is known to hyperactivate tau phosphorylation, leading to its aggregation. Similarly, CDK5, a kinase that phosphorylates tau at multiple NFT-associated sites, has been targeted using indirubin derivatives, with MS studies tracking tau phosphorylation patterns in response to CDK5 inhibition (121, 122). In addition, p38 MAPK inhibitors, such as AZD1080 and MW150, have shown potential in reducing tau phosphorylation and neuroinflammation, further supporting their role as early-stage AD therapeutics (123).

### *N*-Glycosylation

**Fig. 4:**
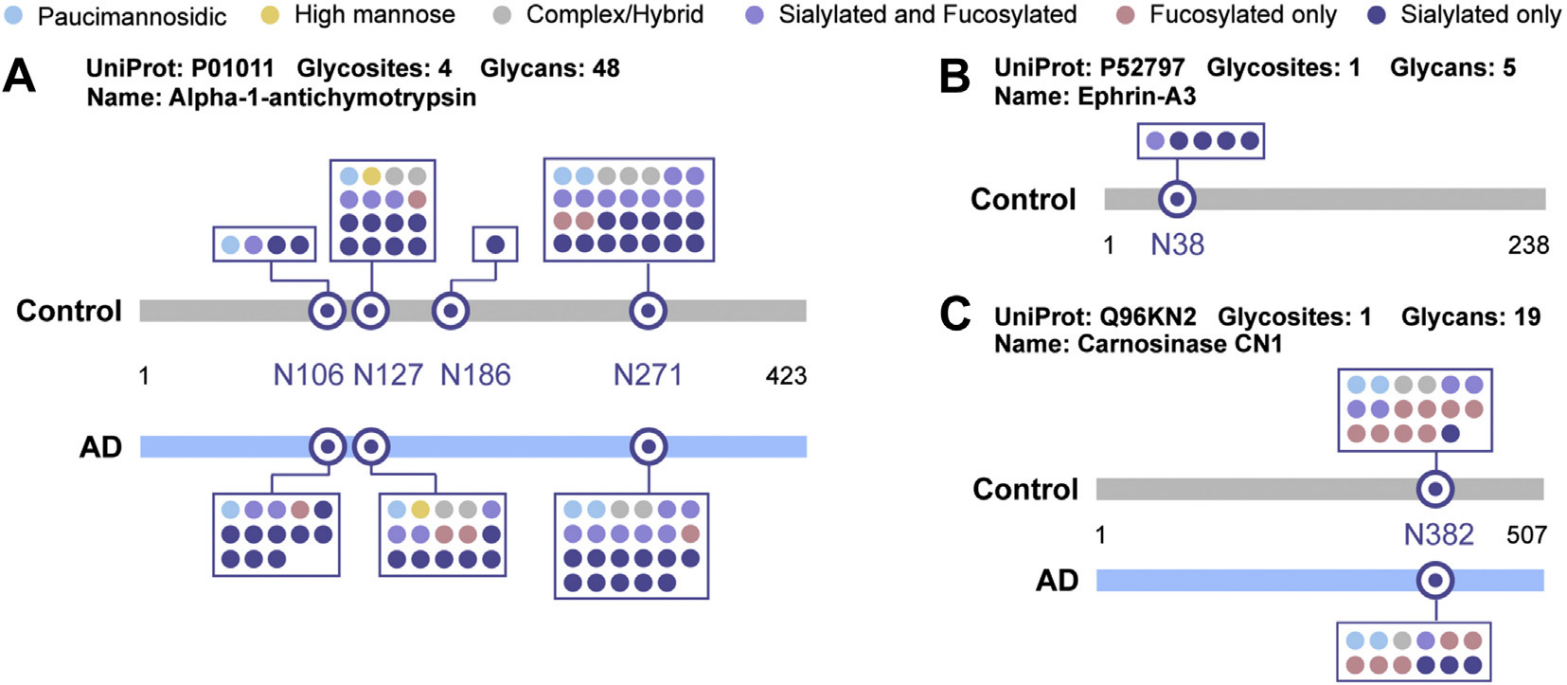
**Site-specific*****N*****-glycosylation analysis.** Glycosylation microheterogeneity for alpha-1-antichymotrypsin (*A*), ephrin-A3 (*B*), and carnosinase CN1 (*C*) detected in CSF between healthy control and AD. Adapted from Chen *et al.* (16) with permission.

The glycosylation of APP is known to influence its processing by secretases, which in turn affects the production of Aβ peptides, a key component of amyloid plaques in AD brains (124). Studies using MS-based approaches have identified specific *N*-glycosylation sites on APP that are critical for its proper trafficking and cleavage. For instance, aberrant glycosylation at Asn467 and Asn496 of APP has been associated with altered Aβ production, contributing to the formation of amyloid plaques (125). MS analysis has also revealed changes in the *N*-glycosylation profiles of other proteins involved in AD, such as BACE1 (88). Alterations in BACE1 glycosylation have been linked to its increased activity, leading to elevated levels of Aβ (70). These findings underscore the importance of *N*-glycosylation in modulating the activity of key proteins in AD and highlight the potential of targeting glycosylation pathways for therapeutic intervention. Furthermore, Chen *et al* demonstrated the site-specific intact *N*-glycopeptide characterization using electron-transfer high-energy collisional induced dissociation (EThcD) and mapped the landscape of glycosylation patterns in AD (Fig. 4) (16). These changes in glycosylation profiles have been proposed as potential biomarkers for early diagnosis and monitoring of disease progression. For example, a decreased fucosylation of carnosinase CN1 in CSF may act as a putative marker for AD (16). Such discoveries pave the way for the development of glycosylation-based biomarkers that could complement existing diagnostic tools for AD. Qi *et al* reported another large-scale and site-specific quantitative *N*-glycoproteome profiling study of human brain tissues in AD and control conditions (126), revealing 13 modules of co-regulated *N*-glycopeptides and glycoproteins, with 6 of these modules being linked to AD phenotypes. Aberrant *N*-glycosylation may affect tau clearance mechanisms, such as lysosomal and proteasomal degradation, further exacerbating its accumulation and toxicity (127). Development of software tools also facilitates the study of glycosylation in AD pathogenesis. pGlyco 2.0 was utilized to investigate intact *N*-glycopeptides in both APP/PS1 mouse models of AD and wild-type mice, leading to the characterization of 3524 intact *N*-glycopeptides (128). Oligo-mannose and fucosylated *N*-glycans were found to be highly expressed in the brains of both mouse models, while *N*-glycosylation of most membrane proteins, including glutamate receptors, was notably down-regulated in APP/PS1 mice (128).

### *O*-Glycosylation

**Fig. 5:**
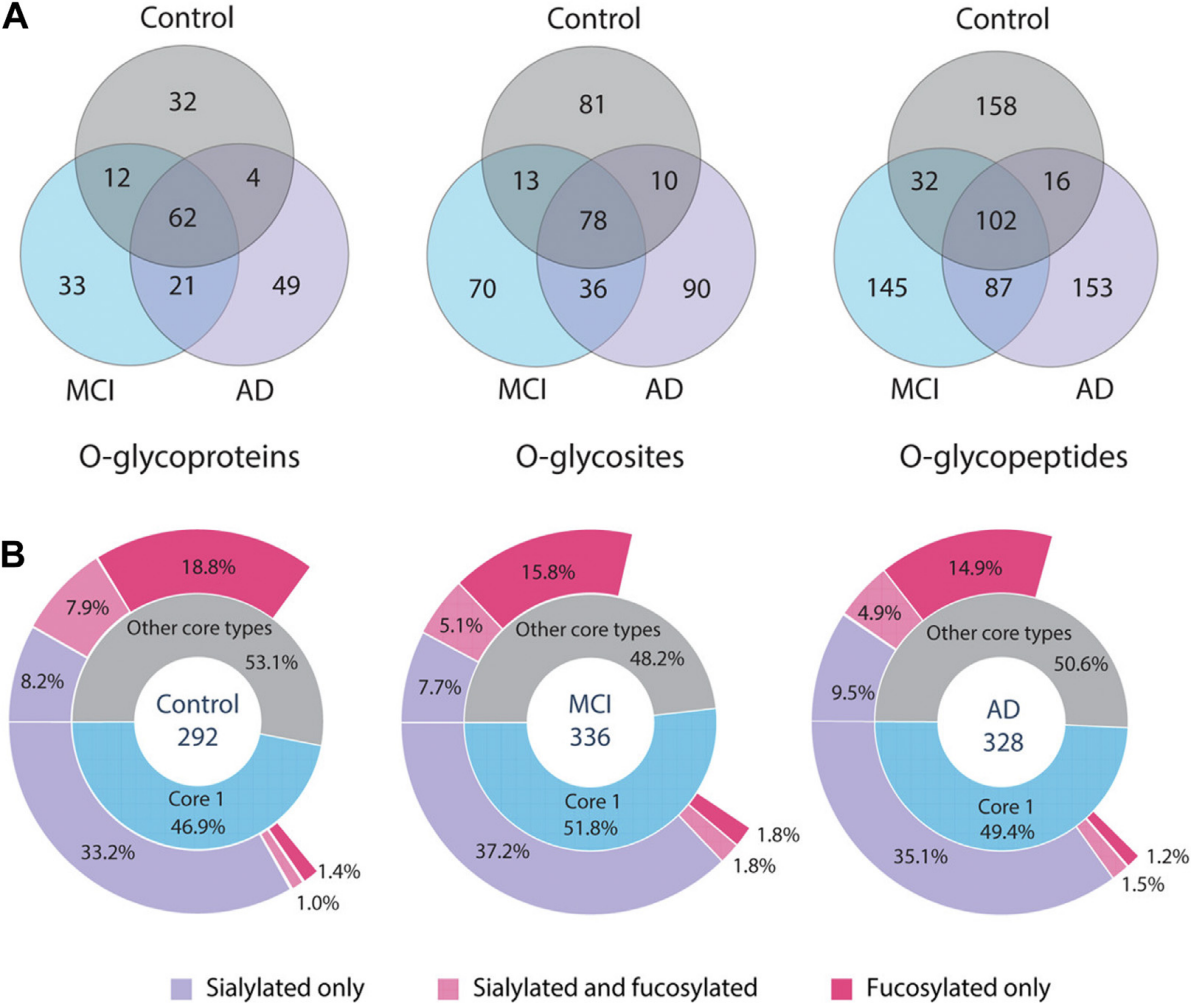
***O*-glycoproteome comparison between control,mild cognitive impairment (MCI), and AD.***A*, Venn diagram analysisof total *O*-glycoproteome in control, MCI, and AD. *B*, comparison of distribution of different types of *O*-glycoforms in control, MCI, and AD. Adapted from Chen *et al.* (15) with permission.

*O*-glycosylation, although less studied than *N*-glycosylation, also plays a crucial role in the pathogenesis of AD. A proteomic analysis of CSF from AD patients has identified a declining trend in fucosylation, alongside an increase in endogenous peptide *O*-glycosylation in the CSF (15) (Fig. 5). Tau protein, which forms neurofibrillary tangles in AD brains, undergoes O-GlcNAcylation at several sites in the cytoplasm (129). MS-based analyses have been instrumental in mapping these *O*-glycosylation sites and understanding their impact on tau aggregation and toxicity. It has been observed that O-GlcNAcylation at Ser400 and Thr403/404 prevents excessive phosphorylation at nearby sites, maintaining tau in a less aggregation-prone state (129, 130, 131). Reduced O-GlcNAcylation in AD brains correlates with increased tau phosphorylation, supporting the hypothesis that O-GlcNAc protects against tau toxicity (132). Consequently, inhibiting OGA, the enzyme responsible for removing O-GlcNAc, has emerged as a promising strategy for treating tau pathology. Recently, the novel selective OGA inhibitor, MK-8719, has progressed to a Phase 1 clinical trial, aiming to restore O-GlcNAc homeostasis and reduce tau pathology (133, 134). In addition to tau, Tyr10-glycosylated Aβ peptides are significantly elevated in the CSF of AD patients, indicating that sialylated *O*-glycans might influence APP processing (135). Another study corroborated that *O*-GalNAcylation at the phenolic hydroxyl group of Tyr681 also impacts APP processing, suggesting that this effect may be due to the conformational changes in APP induced by such *O*-glycosylation (136). More recently, it was observed that hypo-*O*-GalNAcylated APP is externalized to the endothelial cell surface and subsequently transported back to the Golgi apparatus, where it acquires additional *O*-glycans (137). This indicates that the non-classical glycosylation pathway represents a potential novel therapeutic target.

To facilitate the characterization of *O*-glycosylation in AD-related proteins, recent advancements in *O*-glycoproteases have provided powerful analytical tools. Emerging novel *O*-glycoproteases, such as IMPa and StcE, help digest and facilitate the fragmentation and identification of intact *O*-glycosylated peptides (42, 138, 139). These techniques provide more comprehensive fragmentation patterns that allow for the precise localization of *O*-glycosylation sites on proteins. As a result, researchers are now able to study *O*-glycosylation in greater detail, leading to new insights into its role in AD.

Advancements in MS-based techniques have significantly expanded our understanding of the intricate glycosylation patterns involved in AD. Both *N*-glycosylation and *O*-glycosylation play critical roles in the modulation of key proteins such as APP and tau, with direct implications for amyloid plaque formation and neurofibrillary tangle development. These discoveries have opened new avenues for biomarker development and therapeutic interventions, targeting specific glycosylation pathways.

## Citrullination

**Fig. 6:**
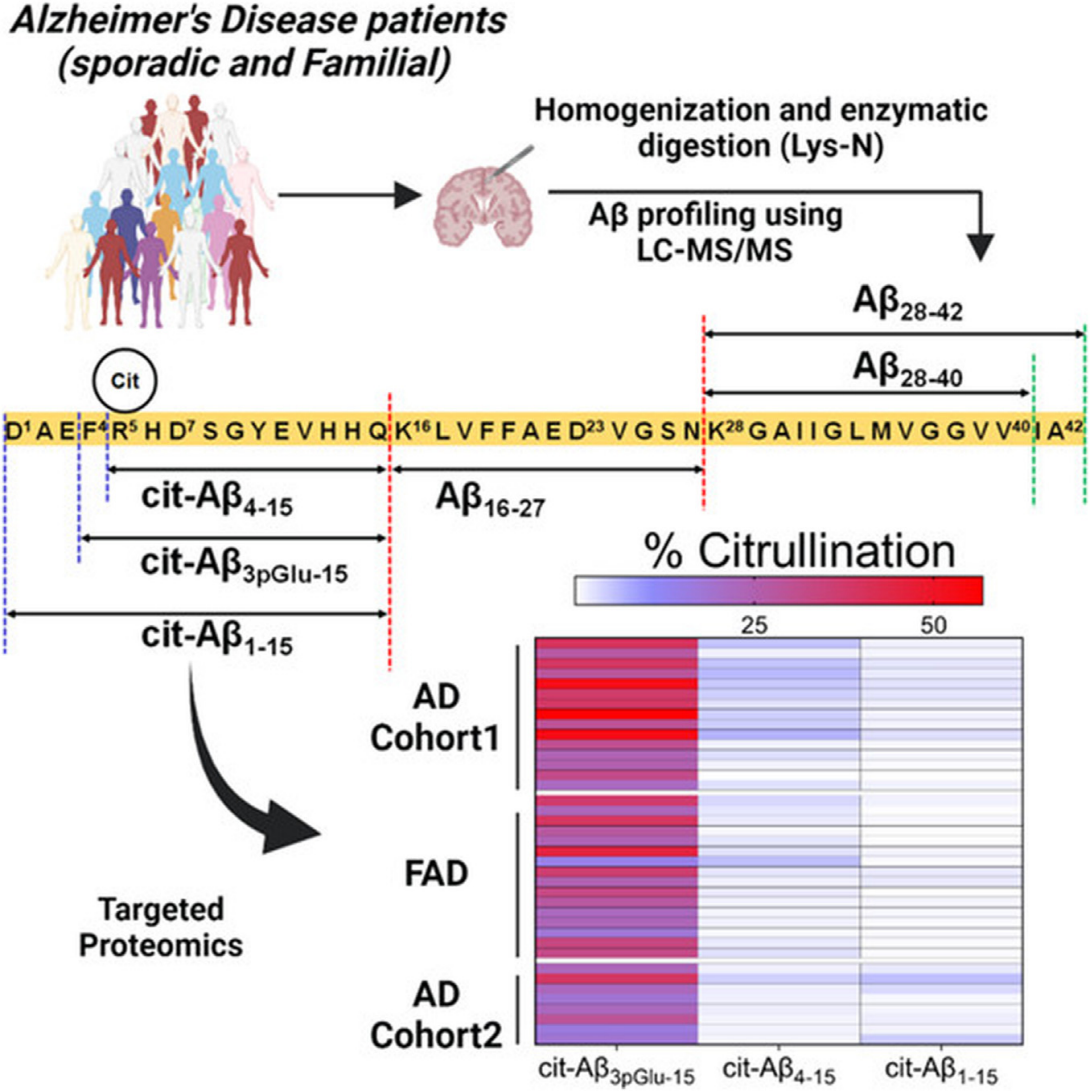
**Identification of Aβ citrullination in sporadic and familial AD brains by characterizing the tandem mass spectra of endogenous N-truncated citrullinated Aβ peptides.** Adapted from Mukherjee *et al.* (165) with permission.

Protein citrullination is catalyzed by a family of enzymes called PADs (140, 141, 142). Due to the guanidine group, arginine has a +1 charge at physiological pH, whereas citrulline has no charge (neutral). This charge neutralization significantly affects protein folding, stability, and interactions, which may contribute to the molecular pathology of AD (143). In some severe cases, dysregulation of protein citrullination can trigger the immune system to generate autoantibodies contributing to many autoimmune processes (143, 144, 145, 146, 147). Numerous studies have reported that the immune system, including innate and adaptive immunity, is emerging as a key player in the pathological process of AD (147, 148, 149). The characteristics of immune cells change dynamically with disease progression, and they shape AD pathology through complex mechanisms. In rheumatoid arthritis, anti-citrullinated protein antibodies detected in patient serum are utilized for early clinical diagnosis (150, 151, 152, 153, 154, 155). Accumulating evidence links dysregulated citrullination to neurodegenerative diseases, notably AD (156, 157, 158, 159, 160). In AD patients, PAD2 and PAD4 enzymes, citrullinated vimentin, and glial fibrillary acidic protein accumulate abnormally in the hippocampus and cortex (161). Inhibiting PADs has emerged as a novel therapeutic strategy. Cl-amidine (PAD inhibitor) has shown reduced citrullinated tau and Aβ pathology in preclinical AD models (162, 163). Citrullination of Aβ42 in AD brains suggests a potential correlation with AD pathology, although this relationship remains insufficiently studied (164). In the context of chronic neuroinflammation and Ca^2+^ dyshomeostasis in AD, understanding the role of microglial activation, gliosis, and Aβ citrullination in both sporadic and familial AD holds significant implications for anti-amyloid immunization therapies. Recently, Mukherjee *et al* revealed that approximately 35% of the pyroglutamate3-Aβ pool was citrullinated in plaques from the temporal cortex of cases with sporadic AD, and about 22% in the detergent-insoluble fractions from the frontal cortex (Fig. 6). In familial AD cases, hypercitrullinated pyroglutamate3-Aβ (∼30%) was observed in both detergent-soluble and detergent-insoluble Aβ pools, contributing to plague persistence and neurotoxicity (165). A comprehensive investigation of citrullination alterations in AD is essential for understanding its pathogenesis and improving diagnostic and therapeutic strategies.

## Other PTMs and Their Contributions to AD Pathology

In addition to the important roles of phosphorylation, glycosylation, and citrullination in AD, other PTMs such as acetylation also contributed to AD pathology. MS studies have identified Lys163/280/281/369 as major acetylation sites on tau, with Lys280 being particularly important for tau pathology. This was demonstrated in Tg mouse brains, where alterations in Lys280 acetylation were linked to disease progression (54, 166). In a *Drosophila* AD model, the pseudo-acetylated tau form (K280Q) results in similar AD-related pathology, expressing the pseudo-acetylated form of tau (K280Q) caused locomotion defects and neurodegeneration in photoreceptor neurons, suggesting that single-site acetylation is sufficient to exacerbate tau-related AD pathology (167). Methylation of protein phosphatase 2A (PP2A) is critical for the dephosphorylation of tau, and this process has been correlated with plasma homocysteine levels in AD (168). Additionally, reductions in PP2A activity, triggered by Aβ and estrogen deficiency, may contribute to the increased risk of AD during menopause (169). Furthermore, protein degradation pathways, particularly SUMOylation and ubiquitination, play essential roles in AD. Impaired clearance mechanisms contribute to the accumulation of pathological aggregates, making SUMOylation and ubiquitination modulation promising therapeutic approaches (170, 171). Strategies to enhance tau degradation and aggregate clearance include Anle138b, a SUMOylation modulator, has demonstrated reduced tau aggregation in AD models (172). MS-based SUMO proteomics enables tracking of tau SUMOylation levels, guiding the development of small-molecule modulators (173, 174).

## Conclusions and Current Challenges

MS-based studies on PTMs in AD have made significant strides in identifying key molecular alterations associated with disease progression. Recent advances in MS have allowed for high-throughput, site-specific characterization of PTMs, including phosphorylation, glycosylation, and citrullination, which play critical roles in the misfolding and aggregation of proteins such as tau and amyloid-beta. These studies have improved our understanding of how PTMs regulate protein function, stability, and interaction networks, shedding light on potential therapeutic targets and early biomarkers for AD. Moreover, novel MS-based methods have enhanced the ability to detect low-abundance modified peptides, contributing to more comprehensive PTM biomarker validation and therapeutic target discovery, paving the way for precision medicine approaches in AD diagnosis and treatment. One of the foremost challenges in studying PTMs in AD is their inherent complexity. Proteins can undergo multiple types of modifications simultaneously, such as phosphorylation, ubiquitination, glycosylation, and acetylation. These PTMs do not act in isolation; they exhibit crosstalk, where one modification influences the occurrence or function of another (175). For example, phosphorylation of tau protein can affect its ubiquitination and subsequent degradation pathways (176). This interplay complicates the analysis, as deciphering the functional consequences of individual PTMs requires not only identifying the modifications but also understanding their interactions. Current MS technologies, while powerful, often struggle with this level of complexity, especially in the context of large-scale proteomic studies where identifying low-abundance PTMs amidst a complex background of more abundant proteins is challenging.

Another significant challenge is the sensitivity and quantification accuracy of MS in detecting PTMs. For example, glycosylation and phosphorylation often occur at low stoichiometry, meaning that only a fraction of the protein population is modified at a given site. This substoichiometric nature, combined with the typically low abundance of modified peptides, makes their detection difficult in MS-based analyses (14, 35, 177). Additionally, the dynamic nature of PTMs, with modifications rapidly added and removed, poses a challenge for accurate quantification (178). Variability in sample preparation, ionization efficiency, and data acquisition can lead to inconsistencies in quantifying PTMs across different samples and experiments (178). Moreover, the modification of peptides can affect their ionization and fragmentation behavior, further complicating the quantitative analysis (179). This variability poses a significant hurdle in correlating specific PTMs with disease states or progression, a crucial step in understanding their role in AD.

AD is a heterogeneous disease, with variations in clinical presentation, progression, and pathology among patients (1). This heterogeneity extends to the molecular level, where different brain regions and cell types may exhibit distinct protein PTM patterns (6, 94). The complex and often overlapping nature of AD with other neurodegenerative diseases further complicates the identification of disease-specific PTMs. Additionally, the post-mortem nature of most brain tissue samples used in studies adds another layer of complexity, as post-mortem degradation can alter PTM states (180, 181). The presence of various cell types in brain tissues, including neurons, glial cells, and microglia, each with distinct proteomes and PTM profiles, poses a significant challenge in isolating and studying disease-relevant modifications.

Despite the advancements in MS technologies, certain limitations persist that hinder comprehensive PTM analysis in AD. The resolution and mass accuracy of MS instruments, while continually improving, are still sometimes insufficient for distinguishing between isobaric PTMs or PTMs that produce similar fragmentation patterns. Additionally, traditional DDA methods used in MS often lead to the preferential selection of more abundant peptides, resulting in the underrepresentation of low-abundance PTMs. Although DIA methods offer a solution to some of these issues, they require extensive spectral libraries and are computationally demanding (182, 183). Furthermore, the requirement for highly specialized software and bioinformatics tools to process and interpret the vast amount of data generated by MS poses another significant challenge, particularly for labs with limited resources.

## Future Perspectives

To address the challenges of sensitivity, resolution, and quantification in PTM studies, future research should focus on the development and adoption of advanced MS technologies and methodologies. Innovations such as ultra-high-resolution mass spectrometers, electron-transfer dissociation (ETD), and improved ion mobility spectrometry could significantly enhance the detection and characterization of PTMs (15, 16, 184, 185, 186, 187, 188, 189, 190). Additionally, the continued refinement of DIA approaches, coupled with machine learning algorithms for data analysis, could improve the quantification accuracy, reduce bias toward abundant peptides, and support clinical practices (191, 192, 193). Combining MS with complementary techniques such as cryo-electron microscopy or nuclear magnetic resonance spectroscopy could provide a more comprehensive understanding of protein PTMs in their native biological context (194, 195).

Given the cellular heterogeneity in the brain, single-cell proteomics represents a promising future direction for PTM studies in AD. This approach would allow the characterization of PTMs at the individual cell level, providing insights into cell-type-specific modification patterns and their role in AD pathology (196). The ability to analyze PTMs in single neurons or glial cells could uncover novel insights into the molecular mechanisms driving AD and identify cell-specific therapeutic targets (197). Additionally, combining single-cell proteomics with spatial proteomics could provide a detailed map of PTM distributions and alterations across different brain regions and their association with disease progression (198).

The integration of multi-omics approaches, including proteomics, transcriptomics, epigenomics, and metabolomics, holds great promise for a more comprehensive understanding of PTMs in AD. By correlating PTM data with gene expression profiles, chromatin modifications, and metabolic changes, researchers can gain a holistic view of the molecular networks underlying AD (199, 200). This system biology approach could help identify key regulatory nodes and pathways that are dysregulated in AD, offering new avenues for therapeutic intervention. Additionally, the integration of longitudinal omics data from patient cohorts could provide insights into the temporal dynamics of PTMs and their role in disease progression, paving the way for the development of PTM-based biomarkers for early diagnosis and monitoring of AD progression or evaluating treatment efficacy (201).

The future of AD treatment may lie in the development of therapeutics that specifically target aberrant PTMs. This could involve designing small molecules, peptides, or antibodies that modulate the activity of enzymes responsible for adding or removing PTMs (e.g., kinases, phosphatases, acetyltransferases). For instance, inhibitors of tau kinases, such as CDK5, could prevent tau hyperphosphorylation and aggregation, offering a potential therapeutic strategy for AD (202). Similarly, targeting PTMs that influence APP processing or Aβ aggregation could help mitigate the formation of toxic species in AD (203). The challenge will be to develop these therapeutics with high specificity and minimal off-target effects, which requires a deep understanding of the PTM landscape and its functional consequences.

## Conflict of Interest

The authors declare that they have no conflicts of interest with the contents of this article.
